# OC_Finder: Osteoclast Segmentation, Counting, and Classification Using Watershed and Deep Learning

**DOI:** 10.3389/fbinf.2022.819570

**Published:** 2022-03-25

**Authors:** Xiao Wang, Mizuho Kittaka, Yilin He, Yiwei Zhang, Yasuyoshi Ueki, Daisuke Kihara

**Affiliations:** ^1^ Department of Computer Science, Purdue University, West Lafayette, IN, United States; ^2^ Department of Biomedical Sciences and Comprehensive Care, Indiana University School of Dentistry, Indianapolis, IN, United States; ^3^ Indiana Center for Musculoskeletal Health, Indiana University School of Medicine, Indianapolis, IN, United States; ^4^ School of Software Engineering, Shandong University, Jinan, China; ^5^ Department of Computer Science, Rensselaer Polytechnic Institute, Troy, NY, United States; ^6^ Department of Biological Sciences, Purdue University, West Lafayette, IN, United States; ^7^ Purdue Cancer Research Institute, Purdue University, West Lafayette, IN, United States

**Keywords:** deep learning, osteoclast segmentation, osteoclast counting, automatic segmentation, open source software

## Abstract

Osteoclasts are multinucleated cells that exclusively resorb bone matrix proteins and minerals on the bone surface. They differentiate from monocyte/macrophage lineage cells in the presence of osteoclastogenic cytokines such as the receptor activator of nuclear factor-κB ligand (RANKL) and are stained positive for tartrate-resistant acid phosphatase (TRAP). *In vitro* osteoclast formation assays are commonly used to assess the capacity of osteoclast precursor cells for differentiating into osteoclasts wherein the number of TRAP-positive multinucleated cells is counted as osteoclasts. Osteoclasts are manually identified on cell culture dishes by human eyes, which is a labor-intensive process. Moreover, the manual procedure is not objective and results in lack of reproducibility. To accelerate the process and reduce the workload for counting the number of osteoclasts, we developed OC_Finder, a fully automated system for identifying osteoclasts in microscopic images. OC_Finder consists of cell image segmentation with a watershed algorithm and cell classification using deep learning. OC_Finder detected osteoclasts differentiated from wild-type and *Sh3bp2* ^*KI/+*^ precursor cells at a 99.4% accuracy for segmentation and at a 98.1% accuracy for classification. The number of osteoclasts classified by OC_Finder was at the same accuracy level with manual counting by a human expert. OC_Finder also showed consistent performance on additional datasets collected with different microscopes with different settings by different operators. Together, successful development of OC_Finder suggests that deep learning is a useful tool to perform prompt and accurate unbiased classification and detection of specific cell types in microscopic images.

## Introduction

Bone homeostasis is maintained with the balance between bone resorption by osteoclasts and bone formation by osteoblasts, which are tightly coordinated with each other (Okamoto et al., 2017). Osteoclasts are highly specialized bone-resorbing cells that are differentiated from monocyte/macrophage lineage cells, and they play a critical role in various physiological events, including bone development, bone repair, and regulation of mineral balance (Schindeler et al., 2008; Okamoto et al., 2017). Excess osteoclast activity will cause bone loss in a variety of pathological conditions such as osteoporosis, rheumatoid arthritis, periodontitis, multiple myeloma, and metastatic cancer. On the other hand, impaired osteoclast activity results in a pathological condition called osteopetrosis characterized by life-threatening bone fragility due to increased bone density (Boyle et al., 2003; Bi et al., 2017). For example, according to the international osteoporosis foundation, it is estimated that approximately 500 million people worldwide suffer from osteoporosis, causing a huge socioeconomic burden.

Because of the biological importance, osteoclasts have been one of the foci in bone biology. *In vitro* osteoclast differentiation is induced by the stimulation of their progenitor cells with macrophage colony-stimulating factor (M-CSF) and the receptor activator of nuclear factor-κB ligand (RANKL) (Boyle et al., 2003; Okamoto et al., 2017). Differentiated osteoclasts are distinguishable from their progenitor cells by their unique characteristics of multinuclearity and positivity for tartrate-resistant acid phosphatase (TRAP) (Boyle et al., 2003). Since the establishment of osteoclast culture methods (Yasuda et al., 1998), *in vitro* osteoclast differentiation assays have been extensively used to quantify and compare the capacity of the progenitor cells for differentiating into osteoclasts. In the assay, the number of TRAP-positive multinucleated cells on culture dishes is manually counted by eyes as osteoclasts by multiple independent examiners. However, the identification of osteoclasts by human eyes does not always secure objectivity and reproducibility. Thus, automated methods for counting osteoclasts have been long awaited.

Here, we developed OC_Finder, a fully automated osteoclast-counting system on microscopic images. OC_Finder identifies and segments cells in microscopic images and classifies each cell image into TRAP + multinucleated osteoclasts and non-osteoclasts. Segmentation is performed with Otsu’s binarization method (Sezgin and Sankur, 2004) combined with morphological opening and the watershed algorithm (Vincent and Soille, 1991; Roerdink and Meijster, 2000). The classification of cell images is performed *via* deep learning, specifically using a convolutional neural network (CNN).

Deep learning has been widely adopted in different biological and medical science areas for classifying cells in the microscope images (Chen et al., 2016; Zhang et al., 2017; Coudray et al., 2018; Habibzadeh et al., 2018; Meng et al., 2018). However, existing methods have some limitations. For most methods, input images need to be manually processed to contain only one cell, or to have cells manually marked to be classified (Zhang et al., 2017; Coudray et al., 2018; Habibzadeh et al., 2018; Meng et al., 2018). For other methods (Chen et al., 2016), multi-modal data need to be prepared as input to help classification. In contrast, in our work, we carefully designed the watershed algorithm to segment cell images, which enabled a fully automated framework for cell detection and classification. Unlike existing segmentation methods (Al-Kofahi et al., 2018; Falk et al., 2019) that need pixel-wise labeling for training, our approach only needs the position of the center of the cells because we perform segmentation in the initial step of the procedure. In CNN, we adopted a teacher–student model (Tarvainen and Valpola, 2017) and image data augmentation techniques for training, which yielded a high accuracy.

There are two recent related works (Cohen-Karlik et al., 2021; Emmanuel et al., 2021) that reported software to detect osteoclasts. The foremost important difference to note is that these two works did not release the datasets they used and their software to the public. Thus, we were unable to compare with their methods, and users will not be able to use their methods either. In contrast, the code and the dataset or our work are fully released to the public so that biologists can use the software. The dataset and the code will also assist computational biologists to develop new methods. In addition, each of the existing works has notable differences from the current work. The work by Cohen-Karlik et al. (2021) used a different neural network framework to detect cells and classify osteoclasts. Their network outputs bounding boxes of cells, while OC_Finder segments the cell region boundaries. Also, judging from the results shown in their article, OC_Finder seems to have higher accuracy with better agreement with human experts. We can also see that OC_Finder would be easier to apply to other types of cells because cell segmentation is performed with an image processing technique that does not need particular training. The second article (Emmanuel et al., 2021) provides a tutorial on how to use a piece of commercial software for identifying osteoclasts. Since the software is for general purpose of cell classification, to use the software, users need to prepare a dataset by manual annotation and train a neural network by themselves using the prepared dataset, which may not be an easy task for biologists. In contrast, OC_Finder is provided with trained networks, which showed high accuracy in the datasets from multiple different microscopes and settings. Thus, it is expected that OC_Finder shows sufficient performance for users without training the network newly from scratch. Also, an automatic segmentation is not achieved in the proposed pipeline. The target of the analysis is also different; the pipeline is for cell identification *in vivo* on histology, while OC_Finder is for osteoclast counting *in vitro*.

OC_Finder achieved 99.4% accuracy in segmentation and 98.1% accuracy in classification. It also achieved 99.5% accuracy in segmentation and 92.9% accuracy in classification for the extra nine datasets collected from different microscopies with different settings by different operators. The number of osteoclasts classified by OC_Finder was at the same level as counting by eye. Together, the successful development of OC_Finder suggests that deep learning is a useful tool for performing prompt and accurate identification and classification of cells with characteristic morphological features in microscopic images with no bias. This approach may be applied to classify non-cellular objects. OC_Finder is available at http://github.com/kiharalab/OC_Finder and https://bit.ly/OC_Finder (online platform). The dataset used in this work is also made freely available at https://doi.org/10.5281/zenodo.5022015, and the nine additional testing datasets are available at https://doi.org/10.5281/zenodo.5822628.

## Materials and Methods

### *In Vitro* Osteoclast Differentiation for Constructing Training and Testing Datasets

**FIGURE 1:**
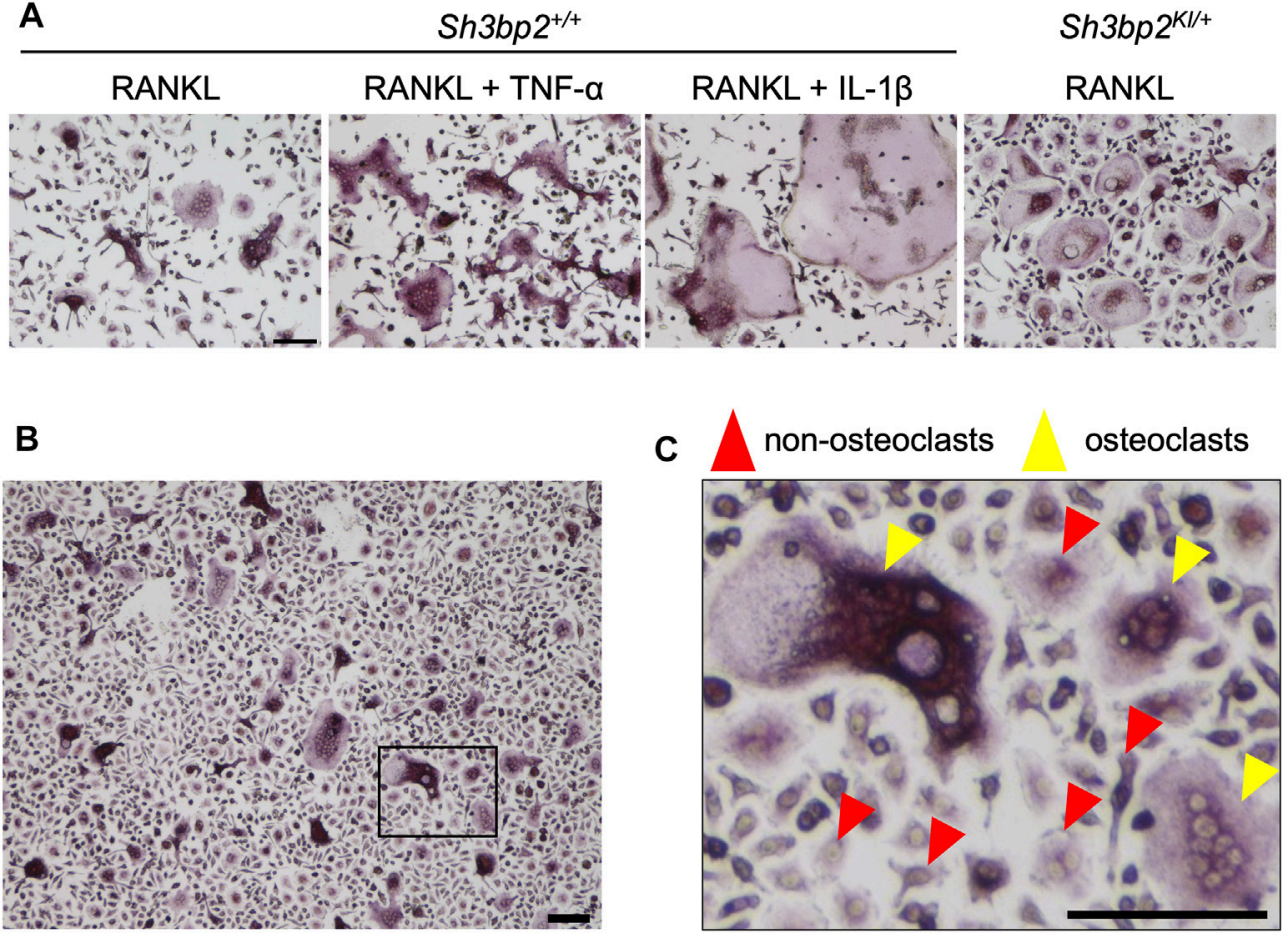
Dataset of cell images of osteoclasts. **(A)** Examples of images of various forms of osteoclasts obtained under different conditions. The concentrations of each cytokine in the culture media were as follows: RANKL: 50 ng/ml, TNF-α: 100 ng/ml, and IL-1β: 10 ng/ml. **(B)** An example of the captured microscopic images of osteoclast culture. **(C)** A magnified image of the boxed area in panel **(B)**, showing the examples of the induced osteoclasts and non-osteoclasts. The cells which are positive for TRAP staining and have more than 3 nuclei were identified as osteoclasts (yellow arrowheads), while all other cells which do not satisfy the criteria were regarded as non-osteoclasts (red arrowheads). Black bar = 100 µm.

Bone marrow cells were isolated from the tibia, femur, and ilium of 7- to 8-week-old male and female *Sh3bp2* ^*+/+*^ and *Sh3bp2* ^*KI/+*^ mice on the C57BL/6 background. The *Sh3bp2* ^*KI/+*^mice that have the heterozygous gain-of-function mutation in SH3 domain-binding protein 2 (SH3BP2) were previously reported (Ueki et al., 2007; Mukai et al., 2014; Kittaka et al., 2020). After treating with red blood cell lysis buffer (eBioscience), bone marrow cells were cultured in alpha-MEM supplemented with 10% FBS and 1% penicillin/streptomycin on the petri dish. After 3 h, non-adherent cells were collected and further cultured in alpha-MEM containing 25 ng/ml M-CSF (PeproTech, East Windsor, NJ) on the petri dish for 3 days to selectively grow the bone marrow-derived M-CSF-dependent macrophages (BMMs). BMMs were harvested, seeded on 48-well plates at a density of 2.5 × 10^4^ cells per well, and cultured for 3 days in the presence of four combinations of cytokines: 1) 25 ng/ml M-CSF and 25 ng/ml RANKL, 2) 25 ng/ml M-CSF and 50 ng/ml RANKL, 3) 25 ng/ml M-CSF and 50 ng/ml RANKL and 100 ng/ml TNF-α, and 4) 25 ng/ml M-CSF and 50 ng/ml RANKL and 10 ng/ml IL-1β. All cytokines were obtained from PeproTech. Osteoclasts exhibit a variety of morphologies depending on the conditions *in vitro*. Therefore, to train the neural network with a variety of osteoclasts in size or morphology, two different BMM sources (*Sh3bp2* ^*+/+*^ and *Sh3bp2* ^*KI/+*^ mice) in the presence of four different cytokine combinations were used. The examples of diverse morphologies of osteoclasts produced in different culture conditions are shown in Figure 1A. Adding TNF-α and IL-1β to the culture induced bigger osteoclasts compared to the osteoclast induction with RANKL only. Gain of function of SH3BP2 by *Sh3bp2* ^*KI/+*^ resulted in an increase in both the number and size of osteoclasts, as previously reported (Ueki et al., 2007), and the induced osteoclasts showed more rounded morphology than the wild-type (*Sh3bp2* ^*+/+*^) control.

### Osteoclast and Non-Osteoclast Image Collection

Cells were stained by tartrate-resistant acid phosphatase (TRAP) staining (Sigma-Aldrich, St. Louis, MO, United States), and images were captured using the BZ-X810 inverted microscope (Keyence, Osaka, Japan) in the bright-field mode with the following settings: ×10 objective lens (Nikon CFI Plan Fluor DL ×10), 1/175 s exposure time, and 50% transmitted light power of 3.7 W LED. Captured images were in the size of 1920 × 1,440 pixels that correspond to 1,451.11 × 1,088.33 µm (1.32 pixel/um) The example image is shown in Figure 1B. TRAP-positive cells containing more than 3 nuclei were considered as osteoclasts.

### Dataset Collection

We obtained 458 microscopic images (314 images from the *Sh3bp2* ^*+/+*^ cell culture and 144 images from the *Sh3bp2* ^*KI/+*^ cell culture) for the training, validation, and test of the neural network. The dataset included the same number of microscopic images, that is, 229 images each, from male and female mice. Osteoclasts and non-osteoclasts were manually identified and distinguished by visual evaluation. The absolute coordinates of each osteoclast and non-osteoclast on the images were provided manually using the “Multi-point” function of ImageJ (Schneider et al., 2012) and used to locate the osteoclasts and non-osteoclasts. Cell images of osteoclasts and non-osteoclasts were cropped based on the obtained coordinates to be used for the training, validation, and test of the neural network.

We generated two datasets from these images. The first dataset was for testing the segmentation accuracy of OC_Finder (the segmentation dataset). The second dataset was for training neural network and examining the classification accuracy of the method (the classification dataset).

For the segmentation dataset, we selected 10 microscopic images of different culture conditions. The culture conditions were as follows: Osteoclast precursors from males or females with the genotype of *Sh3bp2* ^*+/+*^ or *Sh3bp2* ^*KI/+*^ stimulated with 25 or 50 ng/ml of RANKL; and osteoclast precursors from male wild-type mice stimulated with the combination of 50 ng/ml of RANKL with IL-1β (10 ng/ml) or TNF-α (100 ng/ml). In each of the 10 images, we manually counted all the cells. The number of manually identified cells in an image ranged from 445 to 1823 with a total of 10,221.

For the classification dataset, from each of the 458 images we manually identified about 60 cells, only a fraction of cells in an image, so that we could cover a large number of different microscopic images. In total, we located and labeled 13,822 osteoclasts and 13,833 non-osteoclasts. A cell was considered an osteoclast if it is positive for TRAP staining (pink to purple color in Figure 1) and has more than 3 nuclei (Figure 1C, yellow arrowheads) and were considered non-osteoclasts otherwise (Figure 1C, red arrowheads). Among the 458 microscopic images, 373 images (81.4%) were used for training and validation, while the rest (85 images) were used for testing OC_Finder. The 373 images were further split into 298 images (79.9%) for training, which included 9,276 osteoclasts and 9,278 non-osteoclasts, respectively, and 75 images (20.1%) for validation, which included 2,219 osteoclast and 2,226 non-osteoclasts, respectively. The 85 testing images included 2,327 osteoclasts and 2,329 non-osteoclasts, respectively. Cell images in size of 50 × 50 pixels that include an osteoclast or a non-osteoclast were cropped from the dataset images according to the coordination of cells determined by the aforementioned method and used for training, validation, and testing.

### Counting the Number of Osteoclasts

After TRAP staining, nine images were captured (×10 objective lens, 1/175 s exposure time, and 50% transmitted light power) from each well of the osteoclast culture. Osteoclasts in each of the nine images were identified and counted either by visual evaluation or by OC_Finder. To calculate the total number of osteoclasts per culture well (for Figure 6B), the numbers of osteoclasts per image of nine images from the well was averaged and normalized using the size of the area covered by a single image (1.587 mm^2^) and the surface area of the well (0.95 × 10^2^ mm^2^).

### Overall Architecture of OC_Finder

**FIGURE 2:**
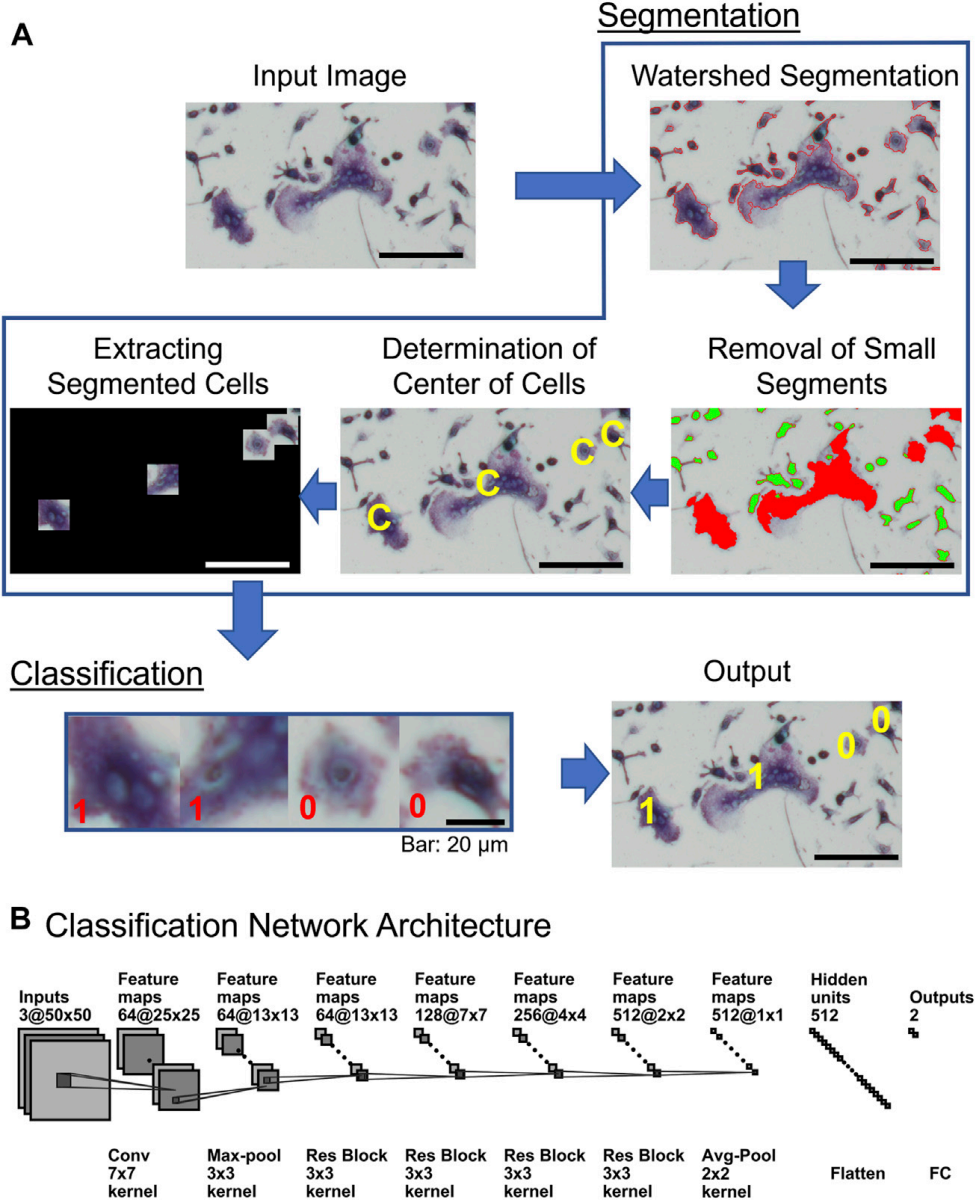
Diagram of OC_Finder. **(A)** The workflow of OC_Finder. First, the input image is processed with Otsu’s binarization, morphology opening and closing, and watershed algorithm to detect the boundary of cells (Watershed Segmentation). After the cells are segmented, small cells with less than 500 pixels (highlighted in green) are removed from further downstream analysis because such cells are never osteoclast (Removal of Small Segments). The center of the remaining segmented regions was computed (Determination of Center of Cells), and then, each cell region is extracted by a square of 50 × 50 pixels that are centered on the acquired cell centers (Extracting Cell Images), which are the input for the deep learning model that classifies it to either as non-osteoclast or osteoclast. Finally, OC_Finder will classify the cropped images to osteoclast and non-osteoclast (Classification) and present the microscopic image with predicted labels assigned to the identified cells as output. **(B)** The deep learning network architecture for cell classification. The architecture is the same as ResNet-18 (He et al., 2016a). Res Block, the residual block, which combines convolution layers, batch normalization, and residual connection (Supplementary Table S2). The notation of the layers, for example, 64@25 × 25 indicates 64 feature maps of 25 × 25 size, and Conv 7 × 7 kernel represents the convolutional operation with a kernel size of 7 × 7. Finally, the network outputs the probabilities that the input cell is non-osteoclast or osteoclast. Bar = 100 µm unless specified in the figure.

OC_Finder processes a given microscopic image with two major steps: segmentation and classification (Figure 2A). First, in the segmentation step, the program identifies cells in the microscopic image and segments them with the watershed algorithm. Next, small cells are removed since they are unlikely to be osteoclasts. Then, the region of each cell is trimmed into the same square image and three colors in the image, RGB, are normalized considering the mean and the variance of the image (Segmentation in Figure 2A). Then, the trained deep learning model is applied to all the trimmed cell images and assign labels (e.g., non-osteoclasts are assigned 0 and osteoclasts are assigned 1 in Figure 2A “Classification”). Finally, OC_Finder visualizes the results with labels assigned to all the segmented cells on the original microscopic image and calculate the number of osteoclasts in the given image. The detailed methods for each step can be found in *Segmentation of cell images* in the “materials and methods” section.

### Segmentation of Cell Images

Cell images were segmented from an input microscopic image using a pipeline that uses the watershed algorithm (Figure 2A, Segmentation) (Vincent and Soille, 1991; Roerdink and Meijster, 2000) as the core of the procedure. We chose this algorithm because it was successful in medical image segmentation tasks (Ng et al., 2006). The procedure started with applying the following preprocessing before applying the watershed. Images were first converted to a grayscale image, then were binarized by using Otsu’s method (Sezgin and Sankur, 2004), which roughly estimates the boundaries between foreground (cell regions) and background. Subsequently, we further applied morphological opening and closing operations to smoothen the cell regions. Otsu’s binarization automatically determines a threshold value to distinguish foreground and background. This algorithm first computes the histogram of grayscales of pixels in an input image. Then, the algorithm applies different intensity thresholds to split the distribution to two distributions. For each threshold, it computes a weighted sum of variance of two distributions and the threshold that yielded the largest sum variance is selected to split foreground and background. This process is performed on-the-fly for each image, and thus no training process is needed. Next, we removed noise in foreground regions by applying morphological opening and closing operations (Vincent, 1994) with a filter of a 3 × 3 pixel size. The opening operation in general removes irregular regions at the boundary, and the closing operation removes holes in foreground regions. Subsequently, we applied a distance transform (Felzenszwalb and Huttenlocher, 2012) to all foreground regions, which tries to separate individual cells from large foreground regions that include multiple cells. In the distance transform, the label of each pixel, which is binary, 0 or 1, at this step due to Otsu’s binarization, was updated to the distance to the closest background pixel. Thus, pixels that are deep inside a cell tend to have a large value. We used 0.7 of the maximum distance in the entire image as the threshold to select pixels as possible cell centers (called markers), which were used as starting points by the watershed algorithm. This procedure was implemented with the OpenCV (Bradski and Kaehler, 2008) package. We used the same setting across all our experiments.

During the development, we have tried Cellpose (Stringer et al., 2021), a recently developed tool for cell image segmentation. However, as shown in (Supplementary Figure S1), it did not perform well on our cell culture images, which include a diverse size of cells in one microscopy image. It only detected the relatively large cells while a large number of small cells, were left undetected, which included osteoclasts (Supplementary Figure S1 yellow arrowhead). Therefore, we decided to develop the original segmentation pipeline.

After segmentation, we removed small segmented regions with less than 500 pixels because they are either a part of a large cell or non-osteoclast for 100% of the cases and would not affect the results of detecting osteoclasts (Figure 2A, Removal of Small Segments). The center of the remaining segmented regions was computed (Figure 2A, Determination of Center of Cells), then, each cell region is extracted by a square of 50 × 50 pixels that are centered on the acquired cell centers (Figure 2A, Extracting Cell Images). These square images are inputs for the cell classification by deep learning.

### Network Architecture

Figure 2B shows the neural network architecture of the cell classification model. The network uses the convolutional neural network (CNN) (Goodfellow et al., 2016), a widely used network architecture for image processing. Due to the shared-weight property of convolutional filters, the CNN can provide translation equivariant outputs, which is referred as the feature map. With this spatial friendly feature, CNN has achieved great success in image processing (He et al., 2016b), video understanding (Xu et al., 2017), object detection, and segmentation (Ren et al., 2015). Among the choices of network architecture of CNN, we used ResNet-18 (He et al., 2016a), which is one of the successful architectures. We also tried to use deeper ResNet networks but did not observe clear improvement (data not shown). An input is a color cell image in RGB with a size of 50 × 50 pixels. In total, 64 convolutional filters of 7 × 7 pixels scan the input with a stride of 2 pixels to capture the local texture pattern of the image. This step results in 64 feature maps of a 25 × 25 size. Subsequently, a max-pooling layer, four residual block layers (Supplementary Figure S2) with 64, 128, 256, and 512 residual blocks, respectively, are applied. Then, the output from the last residual block is processed through an average pooling layer to obtain a feature vector. Finally, the feature vector is flattened and passed to a fully connected (FC) layer with 512 neurons and activated by softmax activation function to produce the probability values that the input cell is non-osteoclast or osteoclast.

### Training the Deep Neural Network

Out of 458 microscopic images, 81.4% (373) were used for training, and the rest 18.6% (85) of them were used for testing. The training set was further split into two parts, 80% (298) used for training and 20% (75) for validation. Thus, the data split was performed with the microscopic images but the classification was performed at the individual extracted cell image level. The number of non-osteoclasts and osteoclasts included in the training, validation, and testing are 9,276/9,278, 2,219/2,226, and 2,327/2,329, respectively, for non-osteoclast/osteoclasts. These cells were manually labeled, and the numbers do not include small cell regions with less than 500 pixels.

RGB values of a pixel in an image in the training set were normalized by computing the Z-score:

$$x_{i}=\frac{x_{i}-\mu_{i}}{\sigma_{i}},$$ (1)

where $x_{i}$ is the i th channel (R, G, or B) of a pixel x , and $\mu_{i}$ and $\sigma_{i}$ are the mean and the variance of channel i in the training set $\sigma_{i}$ , respectively. In the validation and testing stages, we used the same mean and variance values that were taken from the training set. In training, each input cell image was subjected to an augmentation. The type and the magnitude of augmentation were randomly selected.

We used a teacher–student architecture; we used a mean teacher model (Tarvainen and Valpola, 2017) for training the model because it is, in general, effective in avoiding overfitting. The mean teacher model (Tarvainen and Valpola, 2017) updates weights of a teacher model with a moving average of the weights from a sequence of student models as follows:

$$\theta_{t}=\alpha \theta_{t-1}+\left(1-\alpha \right)\theta_{t}^{\prime },$$ (2)

where $\theta_{t}\left(\theta_{t}^{'}\right)$ is the parameters of the teacher (student) model at update step t and α is a smoothing coefficient. We tried different α , as shown in Supplementary Table S1, and set it to 0.999 as it gave the highest accuracy in the validation. We used the teacher model in the evaluation.

Two parameters, regularization parameters of L2 regularization and the learning rate, were optimized with the Adam Optimizer (Kingma and Ba, 2014) for minimizing a cross entropy loss. The regularization parameter values tested were (1e-7, 1e-6, 1e-5, 1e-4, 1e-3, 1e-2, and 1e-1) and the learning rate values tested were (2e-5, 2e-4, 2e-3, 2e-2, and 2e-1, 2). Based on the performance on the validation set, a regularization parameter of 1e-5 and a learning rate of 0.002 performed the best. Under a hyperparameter combination, we generated 100 trained models trained on the training set, which were kept at each epoch. Among them, we selected the model with the aforementioned best hyperparameter combination, which performed the best on the validation set and applied it to the test set. The batch size was set to 256 images and the models were trained for 500 epochs. The training was implemented with PyTorch (Paszke et al., 2019).

### Data Augmentation for Network Training

While training the network, we augmented input cell images by randomly applying one of the 12 image transformations (Cubuk et al., 2019; Cubuk et al., 2020). A magnitude of a transformation was also randomly chosen from a predefined range. We followed AutoAugment (Cubuk et al., 2019) to decide the types and the magnitude range of transformations to apply. The 12 types of transformations and the magnitude range are listed in Supplementary Table S2. Examples of the 12 augmentation types are shown in Supplementary Figure S3. The augmentation process allows a significantly higher amount of trainable data to be derived from the fixed amount of images present in the training dataset. We confirmed that having the augmentation improved the classification performance (Supplementary Table S3).

### Additional Nine Test Datasets

Additional nine datasets were collected from osteoclast cultures that are different from those used for the original dataset. Osteoclast precursors obtained from the male wild-type C57BL/6 mice were stimulated with 50 ng/ml of RANKL for 3 days to induce osteoclast differentiation. Cells were then fixed and stained using the methods described previously. Images were captured by a different person from the one who prepared the original dataset. Three different microscope/camera systems were used with three different settings on each system as listed in Supplementary Table S4. Examples of images from these datasets are shown in Supplementary Figure S4.

### Statistical Analysis

One-way ANOVA with the Tukey–Kramer post hoc test was used for comparison between the groups. For correlation analysis, Pearson’s correlation analysis was used for the samples with Gaussian distribution. For the samples that did not show Gaussian distribution, Spearman’s analysis was used. GraphPad Prism (ver. 7; GraphPad Software, La Jolla, CA) was used for all statistical analyses.

## Results

### Segmentation Results

**FIGURE 3:**
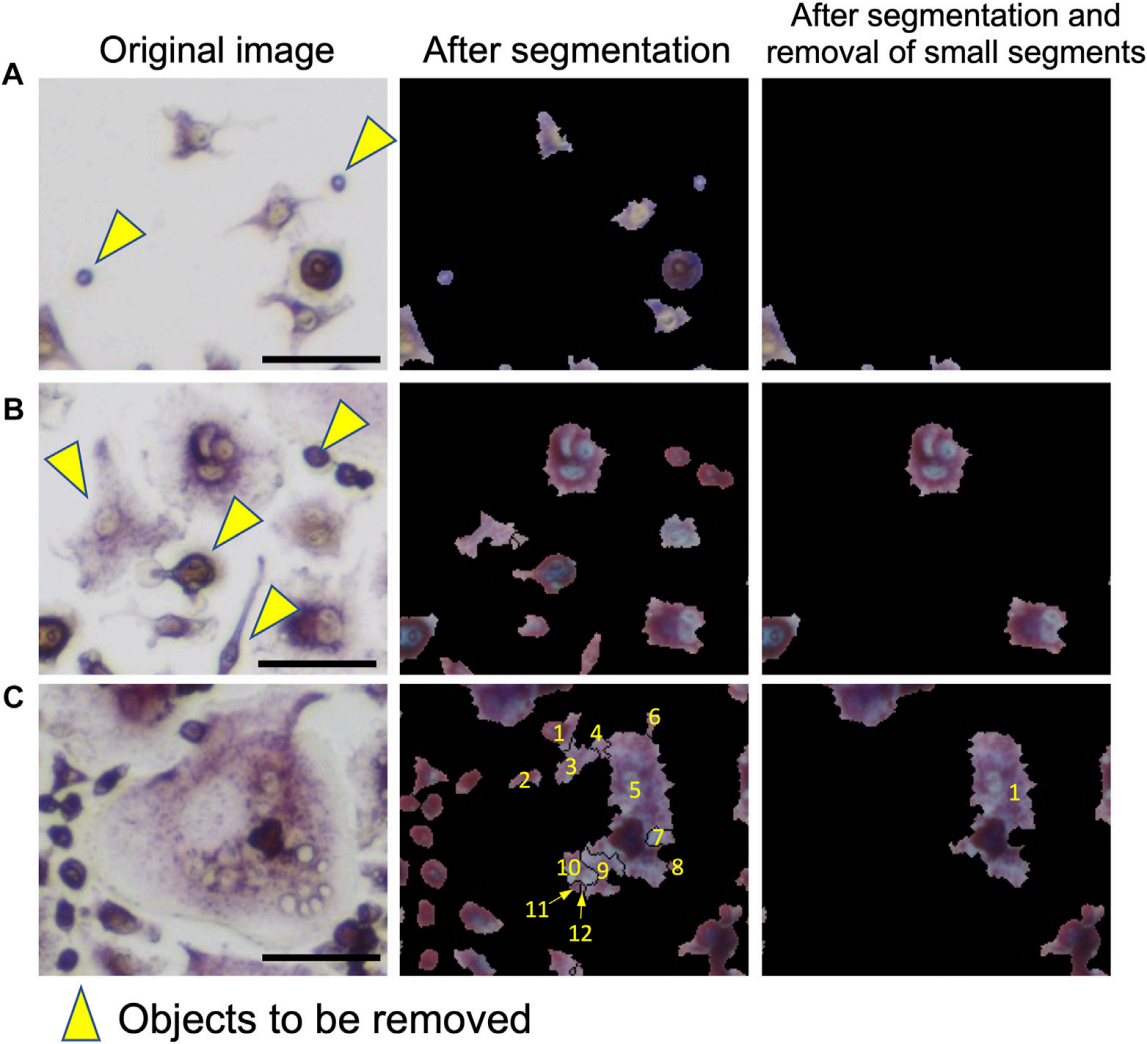
Examples of segmentation and removal of small segments. Three examples are shown. Left: original images. Middle: after segmentation. Right: after the removal of small segments. Objects that were filtered out are pointed out by yellow arrowheads. **(A)** Examples of the removed small debris. **(B)** An example that small regions that correspond to non-osteoclasts were removed by filtering. **(C)** An example where a large cell was segmented into multiple regions. A cell was segmented to 12 areas. Out of them, 11 small regions were removed, leaving only one area remaining. This area was later sufficient to correctly classify this cell as an osteoclast. Bar = 50 µm.

First, we discuss the accuracy of the segmentation step. This step corresponds to “Segmentation” in Figure 2A. Examples of segmentation are shown in Figure 2A and Figure 3. The panels labeled “Input Image” and “Segmentation” in Figure 2A show an example of an input image and a segmentation result. In the “Watershed Segmentation” panel of Figure 2A, the boundaries of segmentations are shown in red, which correspond well to cells in the image. The detailed segmentation results on the 10 microscopic images in the segmentation dataset are provided in Supplementary Table S5. On average, OC_Finder showed a high detection rate of 99.4% of manually detected cells. There were 80 cells that were missed by OC_Finder in the 10 images. Among them, there was only 1 osteoclast included. On the other hand, OC_Finder detected 3 regions that were not included in the manually detected cells. These three regions were not cells but debris, and they are all removed in the subsequent step of the removal of the small regions of less than 500 pixels. The removed debris are shown in Figure 3A (yellow arrowheads).

The step of removing small regions is illustrated in the “Removal of Small Segments” panel in Figure 2A. In the panel, removed segmentations are shown in green, while the remaining large regions are shown in red. Figures 3B,C are additional examples that illustrate the removal of small regions by applying the 500-pixel cutoff. Most of the segmented regions that were removed were non-osteoclasts. Figure 3B shows a part of the microscopic image that includes removed cells (indicated with yellow arrowheads). Figure 3C is an example of a different case, where the removal of small regions helped avoid an overlapped counting of large osteoclasts. In this example, a large osteoclast was segmented into 12 pieces, but 11 of them were removed by filtering, leaving only the largest region, which ultimately allowed it to be correctly classified as an osteoclast.

### Cell Classification Results

Next, we discuss cell classification accuracy. The classification performance was evaluated on the test set of the classification dataset, which includes 2,327 osteoclasts and 2,329 non-osteoclasts, respectively, in 85 microscopic images. The results are summarized in Table 1.

**TABLE 1:** Cell classification accuracy. a) The percentage was computed with two references (denominators). The first percentage was relative to the number of cells that were segmented by the segmentation procedure among all the cells in the classification data set. Thus, 2,179 and 2,308 were used for osteoclast and non-osteoclasts, respectively. The second percentage was computed relative to all the cells in the classification dataset. The percentage values computed in this way were smaller than the former as shown because 3.6% (4,656–4,487) of cells were not correctly segmented and identified by the segmentation procedure.

	Pred. as osteoclast	Pred. as non-osteoclast	Total
All classification test set			
Labels\prediction			
Osteoclast	2,274 (97.7%)	53	2,327 (100%)
Non-osteoclast	35	2,294 (98.5%)	2,329 (100%)
Total	2,309 (98.5%)	2,347 (97.7%)	4,656
After segmentation was applied to the classification test set			
Segmented\prediction			
Osteoclast	2,104 (96.6/90.4%) ^a)^	75	2,179 (100/93.6%) ^a)^
Non-osteoclast	61	2,247 (97.4/96.5%)	2,308 (100/99.1%)
Total	2,165	2,322	4,487 (100/96.4%)

A high classification accuracy, 98.1% (2,274 + 2,294/4,656), was achieved in the classification dataset (Table 1); 97.7% (2,274/2,327) (recall) of the osteoclasts and 98.5% (2,294/2,329) (recall) of the non-osteoclasts were correctly classified. These results were obtained by the teacher model in the teacher–student network we used. In Supplementary Table S1, we compared the current model (α = 0.999) with other models that used different parameter values (for a smoothing coefficient, α. See *Methods*). Particularly, the table shows that the current teacher–student model with *α* = 0.999 performed better than the student model.

Using the classification dataset, we have also evaluated the entire pipeline of OC_Finder, where the segmentation and the classification steps were applied sequentially (Table 1). On this dataset, 96.4% of the cells were segmented correctly, which included 93.6% of the osteoclasts and 99.1% of the non-osteoclasts. 4,351 (2,104 + 2,247) cells, were correctly classified after segmentation. The classification accuracy was 97.0% relative to the 4,487 correctly segmented cells. If all the 4,656 cells in the classification dataset were considered, including the miss-segmented cells, the accuracy would slightly drop to 93.4%.

**FIGURE 4:**
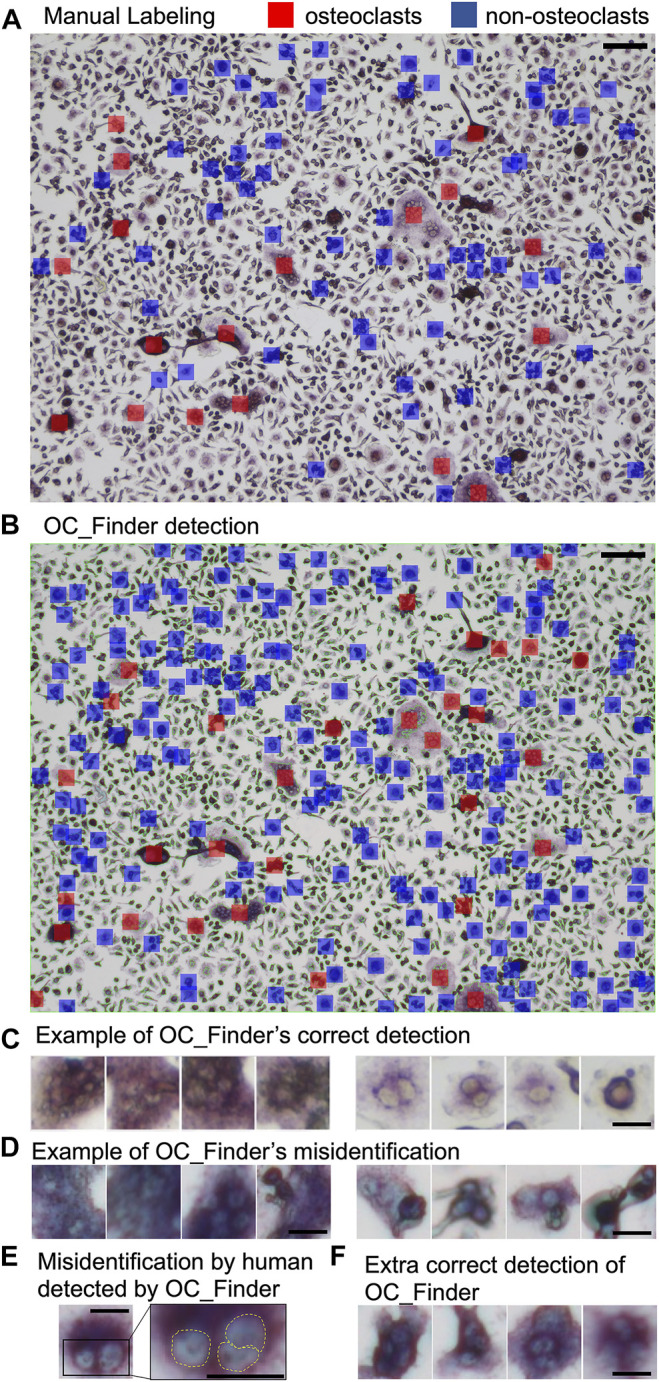
Examples of the validation process for cell classification by OC_Finder. **(A)** Manually labeled osteoclast and non-osteoclasts in a microscopic image. Red indicates osteoclast, and blue indicates non-osteoclast. **(B)** Osteoclasts and non-osteoclasts detected by OC_Finder for the same image. A red box indicates osteoclast, and a blue box indicates non-osteoclast. Cells that are segmented are surrounded by a thin red line. Cells are labeled only when they have a size of 500 pixels or larger. **(C)** Examples of osteoclasts (left) and non-osteoclasts (right) images correctly identified by OC_Finder. **(D)** The examples of osteoclasts (left) and non-osteoclasts (right) images that were misclassified by OC_Finder. **(E)** Osteoclast image that was misclassified by manual annotation but correctly classified by OC_Finder. The right panel is the magnified image of the boxed area in the left panel. **(F)** Examples of osteoclasts that were not picked by the human examiner during the classification dataset construction and identified as osteoclasts by OC_Finder. Bar = 100 µm for **(A)** and **(B)**, 20 µm for **(C–F)**.

Figure 4 shows examples from the validation process for cell classification by OC_Finder. Figure 4A shows manually assigned labels to a microscopic image in the classification dataset. Cells marked with red and blue are osteoclasts and non-osteoclasts, respectively. In the classification dataset, only a part of the cells is manually labeled as mentioned earlier. In Figure 4B, classification results by OC_Finder for this microscopic image is shown. As discussed earlier, small regions were not processed as they are not osteoclasts. The remaining panels contain examples of cells classified by OC_Finder. Figure 4C shows the examples of osteoclasts (four images on the left) and non-osteoclasts (right) that were correctly classified by OC_Finder. One can see that identified osteoclasts were stained by TRAP staining and have more than 3 nuclei, while the non-osteoclasts do not have the properties. Figure 4D shows the opposite, where OC_Finder misclassified cells. On the left, four osteoclasts that were wrongly classified as non-osteoclasts are shown. Nuclei in these cells seem to have unclear boundaries, which might be a reason for the misclassification. The images on the right are non-osteoclasts, which were misclassified as osteoclasts. These four images contain overlapped or adjoining cells that resemble multiple nuclei, which may have confused OC_Finder. Figure 4E is an interesting case where OC_Finder performed better than the human examiner. This cell has three nuclei but the human examiner thought there were only two and thus classified as non-osteoclast since two nuclei are very close to each other and the boundary is not clear. Despite this difficulty, OC_Finder was able to classify it as osteoclast. The last panel (Figure 4F) shows examples where OC_Finder correctly identified osteoclasts from manually unlabeled cells in a microscopic image.

Through this validation process, we confirmed that OC_Finder has a high classification accuracy. We also reaffirmed that humans are prone to error and may occasionally misclassify cell images, in which case OC_Finder can serve as a counteractive measure to human mistakes.

### Test on Nine Additional Datasets

We further tested OC_Finder on nine additional datasets. These datasets are collected by a different person with three different microscopes and 3 settings each (Supplementary Table S4, Supplementary Figure S4). Note that we applied OC_Finder to these datasets without new training or parameter tuning. The detailed results for the individual datasets are provided in Supplementary Tables S6, S7.

**FIGURE 5:**
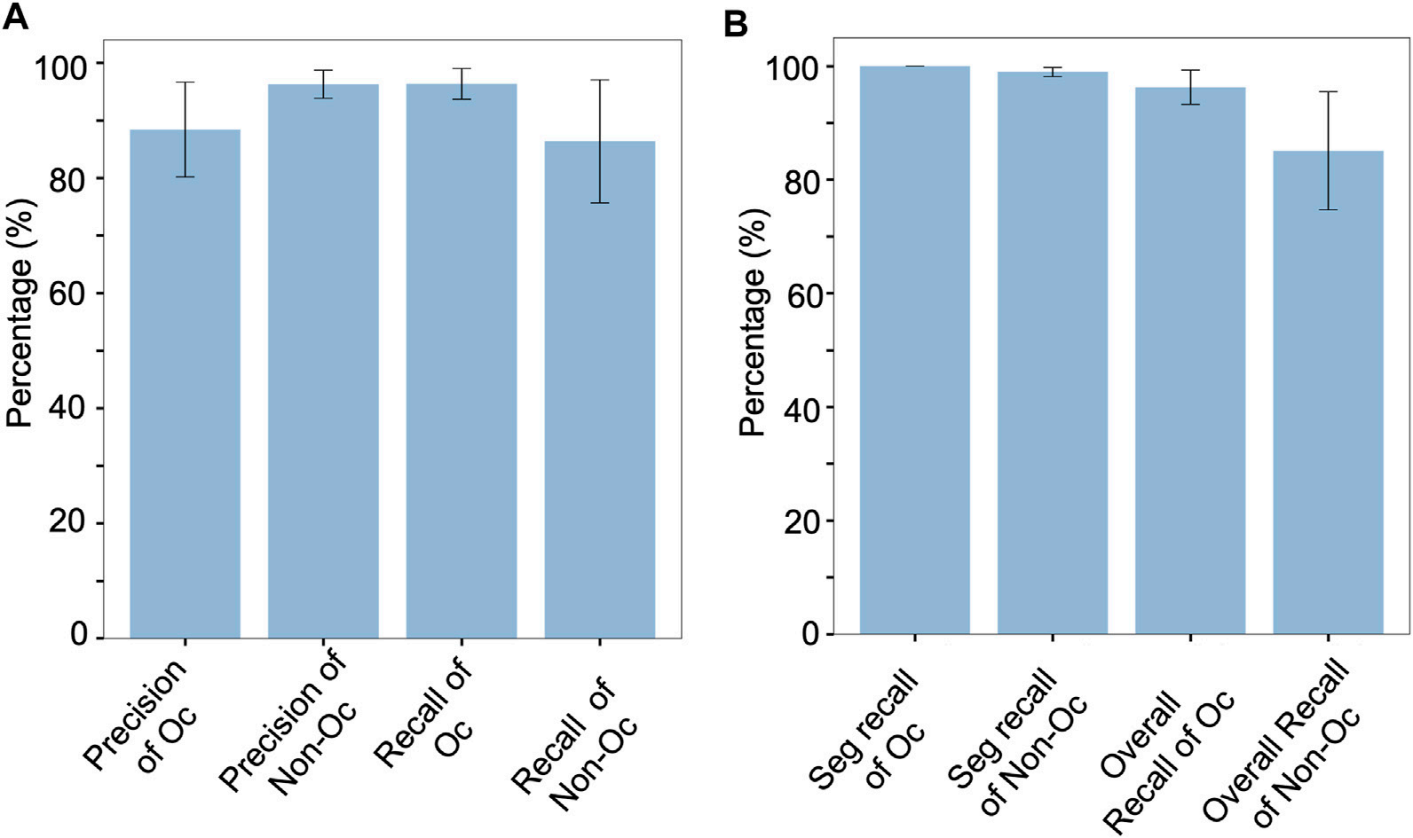
Cell classification accuracy for the nine additional datasets. Values are averaged over the nine datasets. Error bars show standard deviations. Results of the individual dataset are provided in Supplementary Table S7. **(A)** Precision of detecting osteoclasts (Oc), which was computed as the fraction of the number of correctly predicted osteoclasts among all the predicted osteoclasts in a dataset; Precision of non-osteoclasts (non-Oc), computed as the fraction of the number of correctly predicted non-osteoclasts among all the predicted non-osteoclasts in a dataset; recall of Oc, the number of correctly predicted osteoclasts among all the osteoclasts in a dataset; Recall of Non-Oc, the fraction of the number of correctly predicted non-osteoclasts among all the non-osteoclasts in a dataset. The data are taken from part A of tables in Supplementary Table S7. **(B)** Performance of the full pipeline of OC_Finder including the initial cell segmentation step. This graph was generated from part B of tables in Supplementary Table S7. Seg. Recall of Oc, the fraction of correctly segmented osteoclasts among all the osteoclasts in a dataset; Seg. Recall of Non-Oc, the fraction of correctly segmented non-osteoclasts among all the non-osteoclasts in a dataset; Overall Recall of Oc, the fraction of correctly predicted osteoclasts after segmentation among all the osteoclasts in a dataset; Overall Recall of Non-Oc, the fraction of correctly predicted non-osteoclasts after segmentation among all the non-osteoclasts in a dataset.

Segmentation went very well on these datasets with a 98.8% detection rate and no additional segmented regions (Supplementary Table S6). As also illustrated by small error bars in the first 2 bars in Figure 5B, segmentation worked uniformly well for all the nine datasets. Paying attention to cell classification results in Figure 5, we can see that the osteoclasts were classified with a high average recall of 96.4% (Figure 5A, “Recall of Oc”). On these nine datasets, the average precision of classifying the osteoclasts had an average of 88.4%, lower than recall, due to a few datasets that had a relatively low precision of below 90%. When we considered the full OC_Finder pipeline including the segmentation step, the average recall of the detecting osteoclast was still high, 96.3% (Figure 5B, “Overall Recall of Oc”).

Overall, the results on these additional datasets were comparable with the original testing performance reported in Table 1. Particularly, segmentation and recall of osteoclast classification were high and stable. During this work with additional data, there was one case where validation of OC_Finder’s results was not possible because the image was blurred and the manual classification of cells was not possible, although OC_Finder classified such cells (Supplementary Figure S5). Thus, it is noted that the cell images need to have sufficient resolution if a user intends to verify OC_Finder’s results manually.

### Performance of the System in a Practical Situation

**FIGURE 6:**
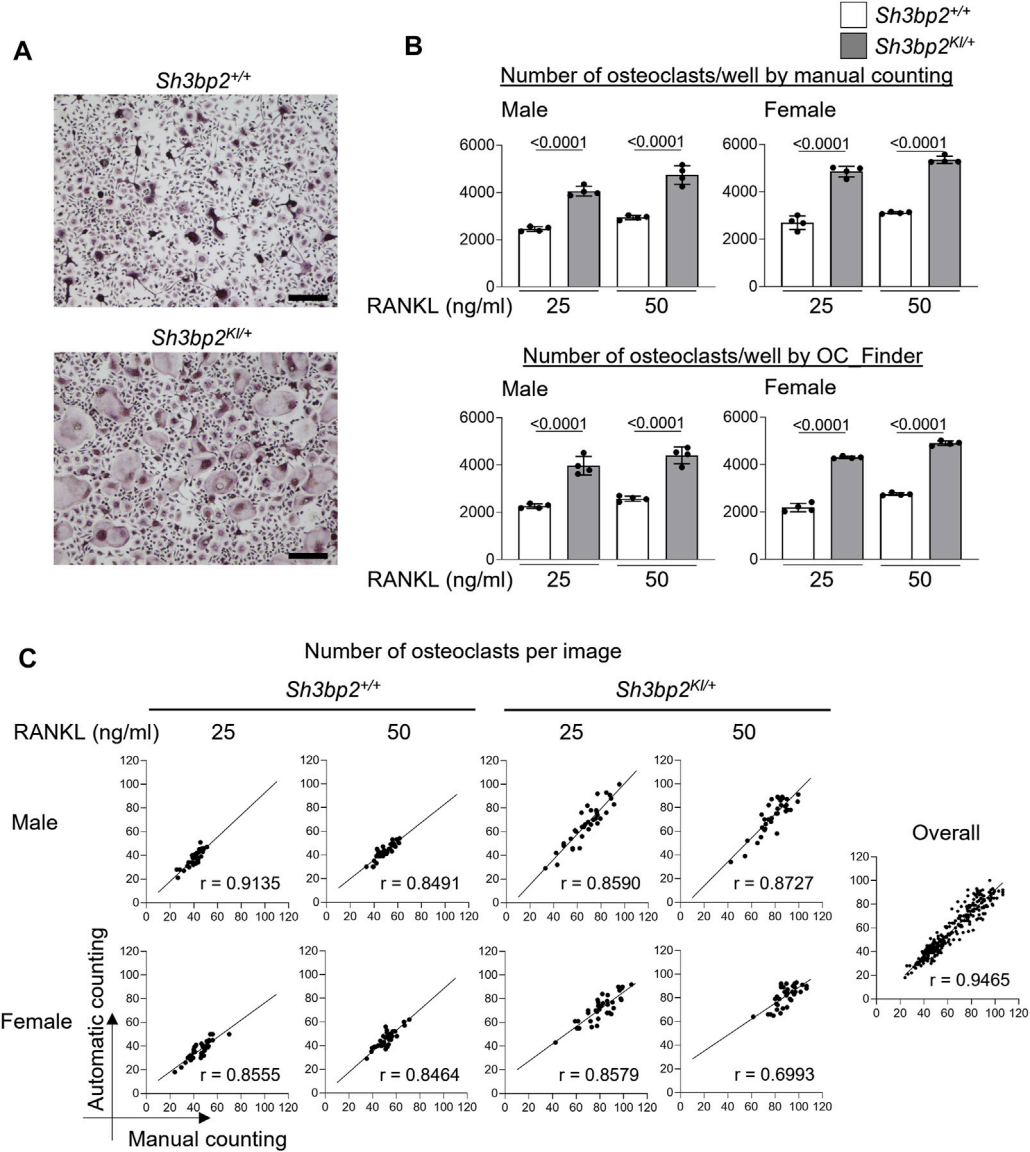
Automatic osteoclast counting compared with manual counting. **(A)** Microscopic images of TRAP-stained osteoclast culture. Upper panel: *Sh3bp2* ^*+/+*^; lower panel: *Sh3bp2* ^*KI/+*^. Bar = 200 μm. **(B)** Validation of the performance of automatic osteoclast-counting in the practical situation. Upper panel: the number of osteoclasts measured manually as reference data. Lower panel: number of osteoclasts measured by OC_Finder. Values on the graphs are *p* values calculated by the Tukey–Kramer test. The *Y*-axis is the number of osteoclasts per culture well, and the *X*-axis is the concentration (25 and 50 ng/ml) of RANKL. **(C)** Correlation analysis between automatic and manual osteoclast counting. The number of osteoclasts on each image was analyzed manually and automatically. In total, 36 images were analyzed for each culture condition. Pearson’s correlation coefficients (r) were shown, except for the analysis for overall samples, which did not show Gaussian distribution and was analyzed with Spearman’s analysis.

Finally, we validated the OC_Finder’s performance in a real-case scenario. Specifically, we examined if OC_Finder could detect an increased osteoclast formation caused by the gain-of-function mutation of *Sh3bp2* (*Sh3bp2* ^*KI/+*^). The gain of function of SH3BP2 is known to increase osteoclast formation (Ueki et al., 2007; Mukai et al., 2014; Kittaka et al., 2020). In this experiment, we included cell sources of different sex (male and female), which is an important factor in fields of biology including bone biology (Rich-Edwards et al., 2018), as well as two concentrations of RANKL (25 and 50 ng/ml) to test whether OC_Finder shows good performance under different experimental designs. Manual counting showed a higher number of osteoclasts in *Sh3bp2* ^*KI/+*^ culture (Figure 6A, the lower panel) than the wild-type control without gain of function of SH3BP2 (*Sh3bp2* ^*+/+*^) (Figure 6A, the upper panel) in the four conditions tested (Figure 6B upper panel, in which the result of *Sh3bp2* ^*+/+*^ and *Sh3bp2* ^*KI/+*^ is presented in white and gray bars, respectively). These results are consistent with the previous reports (Ueki et al., 2007). The automated counting by OC_Finder also detected the difference between *Sh3bp2* ^*+/+*^ and *Sh3bp2* ^*KI/+*^ in all the conditions tested (Figure 6B lower panel) with the same significant *p*-value to the manual counting results.

In Figure 6C, we also compared the number of osteoclasts measured by a human examiner and OC_Finder for each culture condition with 36 microscopic images each. In all the culture conditions, OC_Finder showed a high correlation, between 0.70 and 0.91, with manual counting (Figure 6C left). The correlation was as high as r = 0.9465 when all samples were pooled and analyzed (Figure 6C right). Thus, we confirmed that the automated counting system could generate comparable data to manual counting, and the system demonstrated a good sensitivity to detect biological differences in the experiment.

## Discussion

OC_Finder is the first fully automated osteoclast-counting system that utilizes a deep learning neural network. OC_Finder performs image segmentation and classification tasks in its pipeline. Overall, OC_Finder showed high accuracy in both tasks. When used for a practical scenario of counting osteoclasts (i.e., identifying and classifying cells) in microscopic images, OC_Finder showed comparable performance with human eyes (Figure 6). Therefore, the system can provide valuable assistance in labor-intensive cell counting and greatly reduce the workload for researchers, while maintaining acceptable recall and accuracy.

When the entire pipeline of OC_Finder was applied to microscopic images, the overall accuracy was affected by the segmentation step, which had a slightly lower accuracy than classification. Thus, improvement in segmentation will further increase the system’s accuracy, which is left as a future work. The quality of image segmentation may be controlled by changing parameters, such as the threshold, the filter size, and optimal values, which would be different for different input microscopic images. Users are encouraged to control the parameters for optimizing performance on their own dataset. The code for OC_Finder can be expanded for other similar cell classification tasks by retraining networks on a specific dataset. Expanding the method to handle other cell images is straightforward. OC_Finder will be able to extend to other similar works and be a widely used tool for cell image localization and detection.

## Data Availability

The datasets presented in this study can be found in online repositories. The names of the repository/repositories and accession number(s) can be found below: https://doi.org/10.5281/zenodo.5022015, https://doi.org/10.5281/zenodo.5822628.
