# Supplementary material for: OC_Finder: Osteoclast Segmentation, Counting, and Classification Using Watershed and Deep Learning
Source: Front Bioinform. 2022 Mar 25;2:819570. doi: 10.3389/fbinf.2022.819570 (PMC9038109; doi:10.3389/fbinf.2022.819570)
Supplement: Supplementary file 1 [file DataSheet1.pdf]

Supplementary Material

Supplementary Figure S1.

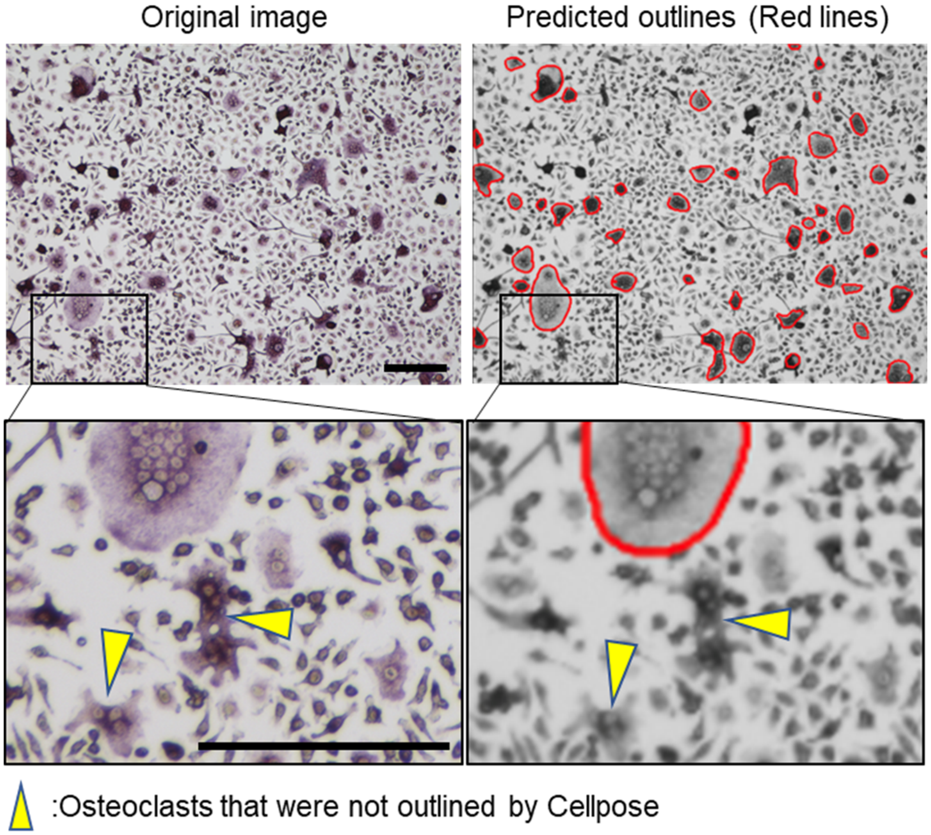

Segmentation performance of Cellpose on our osteoclast culture image. Cellpose outlined only relatively large cells and smaller cells were left not outlined. We ran the Cellpose script with the default setting. Bar = 200 μm.

Supplementary Figure S2.

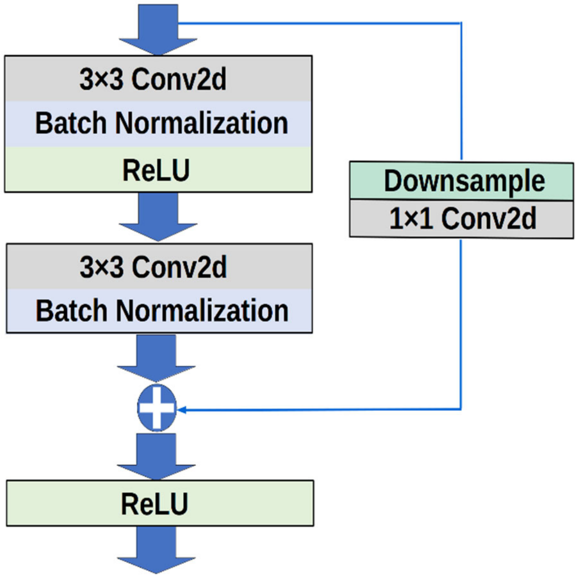

The architecture of the residual block (ResBlock). For a given input image patch, a convolutional filer with size of 3*3, batch normalization, and a ReLU activation are sequentially applied. To further aggregate the spatial information, another convolutional filter and batch normalization are applied for the first output. To avoid the information loss of initial input, a downsample module with a convolutional filter with a size of 1x1 is applied to the initial input to reduce the size of feature map. The network we used has 4 residual layers with 64, 128, 256, and 512 residual blocks, respectively (Figure 1b). Among them, down-sampling is adopted for the last 3 residual layers. If no down-sampling is applied, an identity mapping will apply to the input to output. These two outputs, residual output and the first input, were added and passed to a ReLU activation.

Supplementary Table S1. Accuracy of the Mean-Teacher model with different smoothing coefficient values (α).

EMA α	Training	Validation	Testing
0.0*	99.7	98.3	97.8
0.001	99.7	98.2	97.8
0.005	99.7	98.1	97.8
0.01	99.6	98.0	97.8
0.05	99.6	97.8	97.7
0.1	99.3	97.5	97.5
0.4	99.2	97.3	97.3
0.5	99.4	97.6	97.6
0.8	99.5	97.8	97.6
0.9	99.5	97.9	97.6
0.99	99.6	98.0	97.8
0.999	99.7	98.5	98.1

The classification dataset with 4,654 cells in total was used. The percentage (%) of cells that were correctly classified are reported. α is the parameter in the smoothing coefficient in the Mean-Teacher model, which appears in Eq. 2 in the Method section. $*$ $\alpha = 0.0$ is equivalent with simply using the student model.

Supplementary Table S2. Transformation details for training

Transformation Name	Description	Range
TranslateX(Y)	Translate the image in the horizontal (vertical) direction by magnitude number of pixels.	[-0.3,0.3]
Rotate	Rotate the image magnitude degrees.	[-30,30]
AutoContrast	Maximize the image contrast, by making the darkest pixel black and lightest pixel white.	[0 or 1]
Invert	Invert the pixels of the image	[0 or 1]
Equalize	Equalize the image histogram	[0 or 1]
Solarize	Invert all pixels above a threshold value of magnitude.	[0, 256]
Posterize	Reduce the number of bits for each pixel to magnitude bits	[4,8]
Contrast	Control the contrast of the image. A magnitude=0 gives a gray image, whereas magnitude=1 gives the original image.	[0.1,1.9]
Color	Adjust the color balance of the image, in a manner similar to the controls on a color TV set. A magnitude=0 gives a black & white image, whereas magnitude=1 gives the original image.	[0.1,1.9]
Brightness	Adjust the brightness of the image. A magnitude=0 gives a black image, whereas magnitude=1 gives the original image.	[0.1,1.9]
Sharpeness	Adjust the sharpness of the image. A magnitude=0 gives a blurred image, whereas magnitude=1 gives the original image	[0.1,1.9]

Supplementary Figure S3

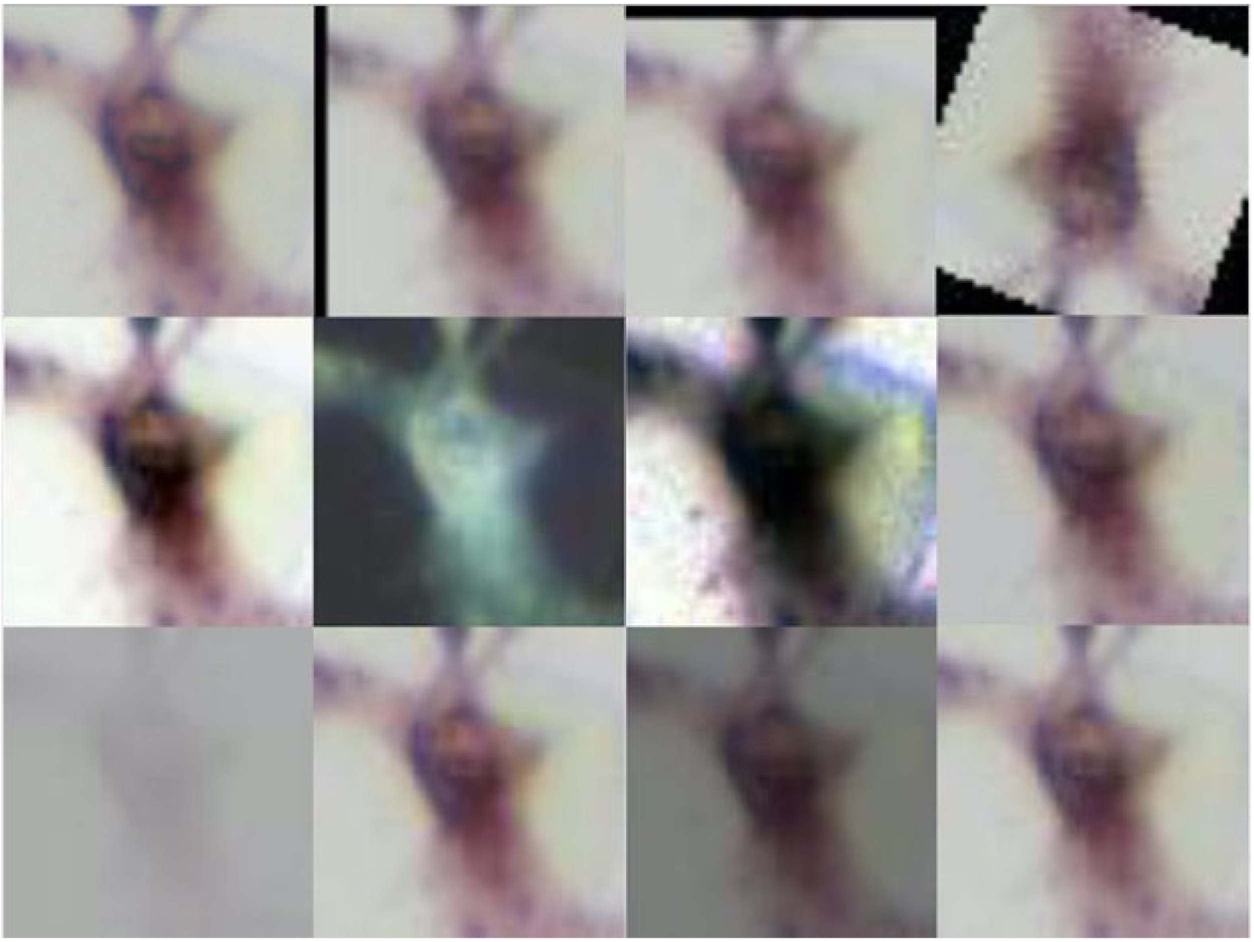

Examples of 12 different augmentations. From the top to bottom rows, left to right: the original image, image with translate-X, translate-Y, rotate, auto-contrast, invert, equalize, solarize, posturize, contrast, color, brightness, and sharpness.

Supplementary Table S3. Accuracy of the model with/without data augmentation

Condition	Training	Validation	Testing
With data augmentation	99.7	98.3	97.8
Without data augmentation	99.8	96.7	96.4
**Total Number images**	9,276+9,278	2,219+2,226	2,327+2,329

The classification dataset includes 458 microscopic images. Among them, 373 images (81.4%) were used for training and validation while the rest (85 images) were used for testing OC_Finder. The 373 images were further split into 298 images (79.9%) for training, which included 9,276 osteoclasts and 9,278 non-osteoclasts, respectively, and 75 images (20.1%) for validation, which included 2,219 osteoclast and 2,226 non-osteoclasts, respectively. The 85 testing images included 2,327 osteoclasts and 2,329 non-osteoclasts, respectively. The percentage (%) of cells that were correctly classified are reported.

With augmentation, accuracy improved on both validation and testing datasets.

Supplementary Table S4. Detailed imaging settings for the nine additional datasets.

Dataset	# of images	Microscope/camera system	Lens	Image size (pixel)	Image size (µm)	pixel/µm ratio	resize	exposure time
#1	18	Keyence BZ-X810	Nikon CFI Plan Fluor DL 10x	1920 x 1440	1451.11 x 1088.33	1.323	none	1/200 s
#2	18	Keyence BZ-X810	Nikon CFI Plan Fluor DL 10x	1920 x 1440	1451.11 x 1088.33	1.323	none	1/120 s
#3	18	Keyence BZ-X810	Nikon CFI Plan Fluor DL 10x	1920 x 1440	1451.11 x 1088.33	1.323	none	1/250 s
#4	36	Leica DMIL LED/Amscope MU1000-HS	Leica HI Plan 1 10x Ph1	1832 x 1374	761.97 x 571.48	2.404	1008 x 756	auto exposure with exposure target 100
#5	36	Leica DMIL LED/Amscope MU1000-HS	Leica HI Plan 1 10x Ph1	1832 x 1374	761.97 x 571.48	2.404	1008 x 756	auto exposure with exposure target 130
#6	36	Leica DMIL LED/Amscope MU1000-HS	Leica HI Plan 1 10x Ph1	1832 x 1374	761.97 x 571.48	2.404	1008 x 756	auto exposure with exposure target 70
#7	16	Echo Rebel	Echo 10x Plan Achromat phase	2732 x 1908	1600 x 1117.67	1.708	1958 x 1478	auto exposure with brightness 50
#8	16	Echo Rebel	Echo 10x Plan Achromat phase	2732 x 1908	1600 x 1117.67	1.708	1958 x 1478	auto exposure with brightness 60
#9	16	Echo Rebel	Echo 10x Plan Achromat phase	2732 x 1908	1600 x 1117.67	1.708	1958 x 1478	auto exposure with brightness 40

We prepared nine datasets using three different microscope/camera systems and three different settings on each microscope system. The images in datasets #4 to #9 were different from our default image size, therefore, they were resized as indicated above before the experiment. The resize function is also included in OC_Finder as an option. These datasets were prepared by a different person (Y. Ueki) from the person (M. Kittaka) who prepared the training set used for the training and the initial evaluation of OC_Finder reported in Table 1.

Supplementary Figure S4.

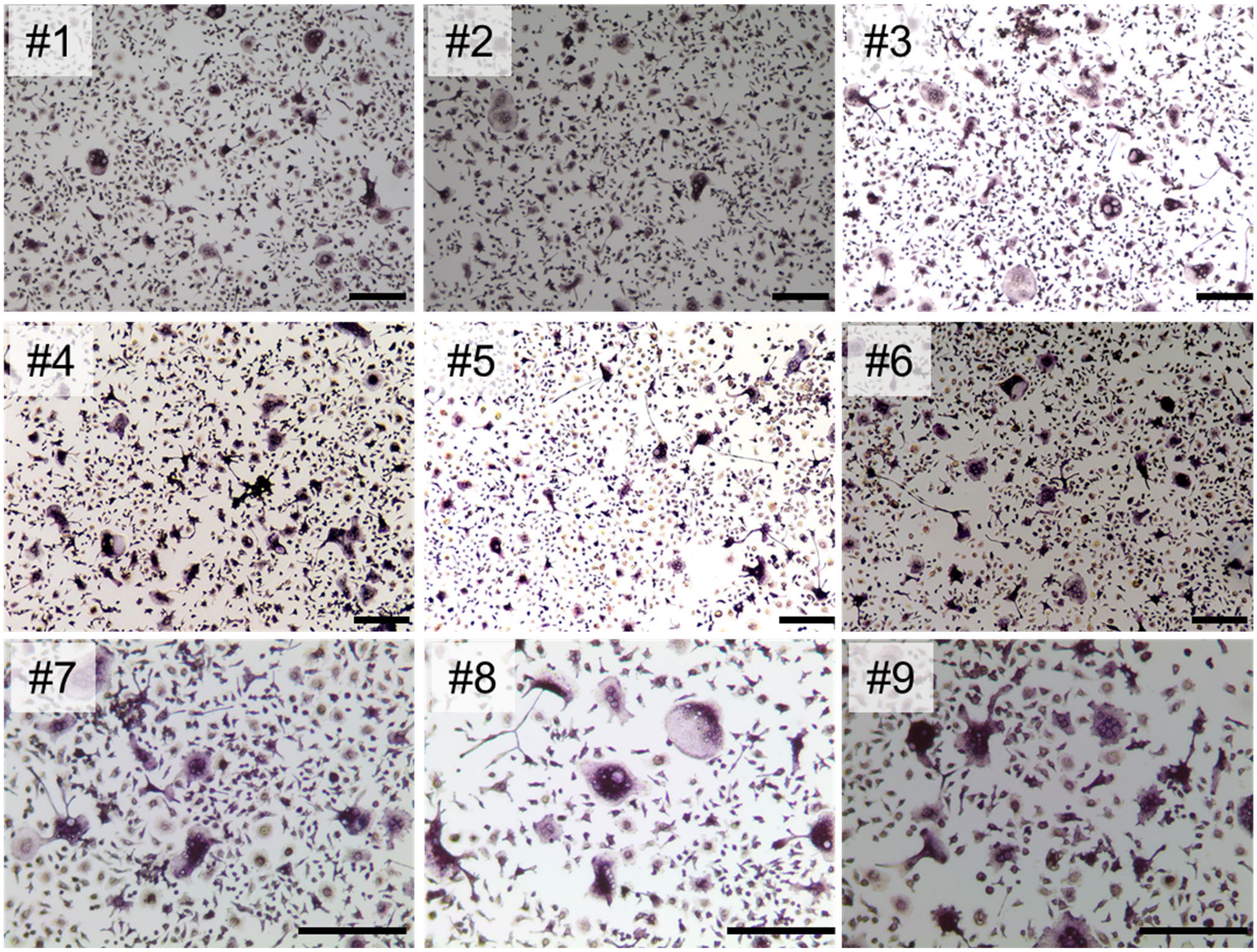

Example images of each of the nine additional datasets used in Figure 5. Detailed information of the system setting is provided in Supplementary Table S4. Bar = 200 μm.

Supplementary Table S5. Cell segmentation results on the 10 microscopic images.

Image	Total	Not Identified	Detection Rate (%)	Additional Detections by OC-Finder
Female *Sh3bp2*$^{+/+}$ stimulated with RANKL (25 ng/ml)	1723	22	98.7	0
Female *Sh3bp2*$^{+/+}$ stimulated with RANKL (50 ng/ml)	1823	17	99.1	0
Female *Sh3bp2*$^{KI/+}$ stimulated with RANKL (25 ng/ml)	1014	5	99.5	0
Female *Sh3bp2*$^{KI/+}$ stimulated with RANKL (50 ng/ml)	883	6	99.3	0
Male *Sh3bp2*$^{+/+}$ stimulated with RANKL (25 ng/ml)	1053	18	98.3	0
Male *Sh3bp2*$^{+/+}$ stimulated with RANKL (50 ng/ml)	1204	7	99.4	0
Male *Sh3bp2*$^{KI/+}$ stimulated with RANKL (25 ng/ml)	445	0	100.0	3
Male *Sh3bp2*$^{KI/+}$ stimulated with RANKL (50 ng/ml)	664	2	99.7	0
Male *Sh3bp2*$^{+/+}$ stimulated with RANKL (50 ng/ml) and TNF-α	635	3	99.5	0
Male *Sh3bp2*$^{+/+}$ stimulated with RANKL (50 ng/ml) and IL-1β	777	0	100.0	0
Average	1022.1	8	99.4	0.3

Cell segmentation by OC_Finder was compared with manually identified cells in 10 microscopic images. The image column lists specification of each images. Total, the total number of cells that were manually identified in each image data. Not identified, the number of cells that were not detected by OC_Finder. Detection rate was computed by (Total – Not_Identified)/Total * 100(%). Additional detection by OC_Finder, the number of additional segments OC_Finder identified that did not correspond to manual identification.

Supplementary Table S6. Cell segmentation results for the additional nine datasets.

Dataset	Total	Not identified	Detection Rate (%)	Additional detection by OC_Finder
#1	1539	14	99.09	0
#2	1437	5	99.65	0
#3	1557	25	98.39	0
#4	1291	8	99.38	0
#5	1420	19	98.66	0
#6	1092	4	99.63	0
#7	1632	13	99.20	0
#8	1376	40	97.09	0
#9	1120	21	98.13	0
Average	1384.89	16.56	98.80	0

Cell segmentation by OC_Finder was compared with manually identified cells in images from each dataset. Total: the total number of cells that were manually identified. Not identified: the number of cells that were not segmented by OC_Finder. Detection rate was computed by (Total – Not_Identified)/Total * 100 (%). Additional detection by OC_Finder, the number of additional segments OC_Finder identified that did not correspond to manual identification.

Supplementary Table S7. Detailed results for the nine additional datasets.

Dataset 1

A. All classification test set.

Labels\Prediction	Pred. as Osteoclast	Pred. as non-Osteoclast	Total
Osteoclast	672 (94.4%)	40	712 (100%)
Non-osteoclast	8	708 (98.9%)	716 (100%)
Total	680 (98.8%)	748 (94.7%)	1,428

B. After segmentation was applied to the classification test set.

Segmented\Prediction	Pred. as Osteoclast	Pred. as non-Osteoclast	Total
Osteoclast	688 (96.6/96.6%) [a)]	24	712 (100/100%) [a)]
Non-osteoclast	12	698 (98.3/97.5%)	710 (100/99.2%)
Total	700	722	1,422 (100/99.6%)

Dataset 2

A. All classification test set.

Labels\Prediction	Pred. as Osteoclast	Pred. as non-Osteoclast	Total
Osteoclast	633 (97.2%)	18	651 (100%)
Non-osteoclast	30	621 (95.4%)	651 (100%)
Total	663 (95.5%)	639 (97.2%)	1,302

B. After segmentation was applied to the classification test set.

Segmented\Prediction	Pred. as Osteoclast	Pred. as non-Osteoclast	Total
Osteoclast	626 (96.2/96.2%) [a)]	25	651 (100/100%) [a)]
Non-osteoclast	40	608 (93.8/93.4%)	648 (100/99.5%)
Total	666	633	1,299 (100/99.8%)

Dataset 3

A. All classification test set.

Labels\Prediction	Pred. as Osteoclast	Pred. as non-Osteoclast	Total
Osteoclast	595 (91.3%)	57	652 (100%)
Non-osteoclast	2	657 (99.7%)	659 (100%)
Total	597 (99.7%)	714 (92.0%)	1,311

B. After segmentation was applied to the classification test set.

Segmented\Prediction	Pred. as Osteoclast	Pred. as non-Osteoclast	Total
Osteoclast	590 (90.5/90.5%) [a)]	62	652 (100/100%) [a)]
Non-osteoclast	5	651 (99.2/98.8%)	656 (100/99.5%)
Total	595	713	1,308 (100/99.8%)

Dataset 4

A. All classification test set.

Labels\Prediction	Pred. as Osteoclast	Pred. as non-Osteoclast	Total
Osteoclast	439 (96.3%)	17	456 (100%)
Non-osteoclast	79	387 (83.0%)	466 (100%)
Total	518 (84.7%)	404 (95.8%)	922

B. After segmentation was applied to the classification test set.

Segmented\Prediction	Pred. as Osteoclast	Pred. as non-Osteoclast	Total
Osteoclast	439 (96.3/96.3%) [a)]	17	456 (100/100%) [a)]
Non-osteoclast	84	380 (81.9/81.5%)	464 (100/99.6%)
Total	523	397	920 (100/99.8%)

Dataset 5

A. All classification test set.

Labels\Prediction	Pred. as Osteoclast	Pred. as non-Osteoclast	Total
Osteoclast	424 (93.0%)	32	456 (100%)
Non-osteoclast	50	416 (89.3%)	466 (100%)
Total	474 (89.5%)	448 (92.9%)	922

B. After segmentation was applied to the classification test set.

Segmented\Prediction	Pred. as Osteoclast	Pred. as non-Osteoclast	Total
Osteoclast	418 (91.7/91.7%) [a)]	38	456 (100/100%) [a)]
Non-osteoclast	58	399 (87.3/85.6%)	457 (100/98.1%)
Total	476	437	913 (100/99.0%)

Dataset 6

A. All classification test set.

Labels\Prediction	Pred. as Osteoclast	Pred. as non-Osteoclast	Total
Osteoclast	451 (98.9%)	5	456 (100%)
Non-osteoclast	138	328 (70.4%)	466 (100%)
Total	589 (76.6%)	333 (98.5%)	922

B. After segmentation was applied to the classification test set.

Segmented\Prediction	Pred. as Osteoclast	Pred. as non-Osteoclast	Total
Osteoclast	451 (98.9/98.9%) [a)]	5	456 (100/100%) [a)]
Non-osteoclast	143	322 (69.2/69.1%)	465 (100/99.8%)
Total	594	327	921 (100/99.9%)

Dataset 7

A. All classification test set.

Labels\Prediction	Pred. as Osteoclast	Pred. as non-Osteoclast	Total
Osteoclast	529 (98.7%)	7	536 (100%)
Non-osteoclast	89	449 (83.5%)	538 (100%)
Total	618 (85.6%)	456 (98.5%)	1,074

B. After segmentation was applied to the classification test set.

Segmented\Prediction	Pred. as Osteoclast	Pred. as non-Osteoclast	Total
Osteoclast	524 (97.8/97.8%) [a)]	12	536 (100/100%) [a)]
Non-osteoclast	79	446 (85.0/82.9%)	525 (100/97.6%)
Total	603	458	1,061 (100/98.8%)

Dataset 8

A. All classification test set.

Labels\Prediction	Pred. as Osteoclast	Pred. as non-Osteoclast	Total
Osteoclast	506 (98.3%)	9	515 (100%)
Non-osteoclast	59	460 (88.6%)	519 (100%)
Total	565 (89.6%)	469 (98.1%)	1,034

B. After segmentation was applied to the classification test set.

Segmented\Prediction	Pred. as Osteoclast	Pred. as non-Osteoclast	Total
Osteoclast	508 (98.6/98.6%) [a)]	7	515 (100/100%) [a)]
Non-osteoclast	49	459 (90.4/88.4%)	508 (100/97.9%)
Total	557	466	1,023 (100/98.9%)

Dataset 9

A. All classification test set.

Labels\Prediction	Pred. as Osteoclast	Pred. as non-Osteoclast	Total
Osteoclast	511 (99.2%)	4	515 (100%)
Non-osteoclast	164	355 (68.4%)	519 (100%)
Total	675 (75.7%)	359 (98.9%)	1,034

B. After segmentation was applied to the classification test set.

Segmented\Prediction	Pred. as Osteoclast	Pred. as non-Osteoclast	Total
Osteoclast	514 (99.8/99.8%) [a)]	1	515 (100/100%) [a)]
Non-osteoclast	161	355 (68.8/68.4%)	516 (100/99.4%)
Total	675	356	1,031(100/99.7%)

Supplementary Figure S5.

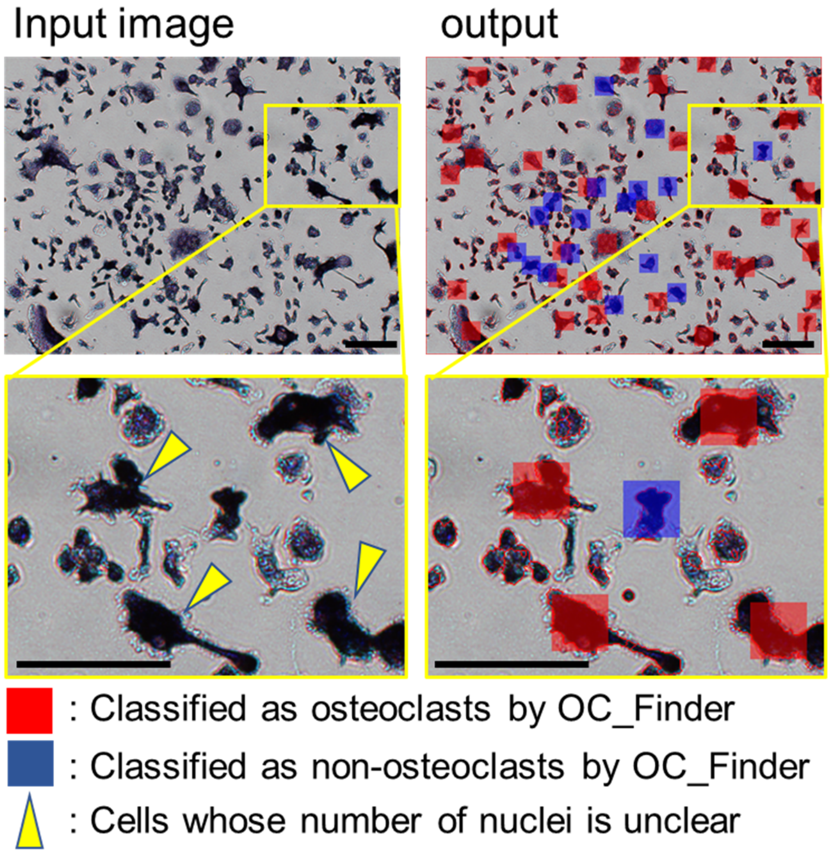

An example of improper images for the use of OC_Finder. Although OC_Finder seemed to properly perform segmentation and classification, it was challenging for human examiners to validate the classification by OC_Finder if they could not see the details of cells such as number of nuclei. Bar = 100 µm. The images were captured using Lionheart FX automated microscope (Biotek) with Olympus UPLFN 10x Ph lens and color bright field mode with fixed default setting. (We did not use this image because we cannot compute the accuracy).
